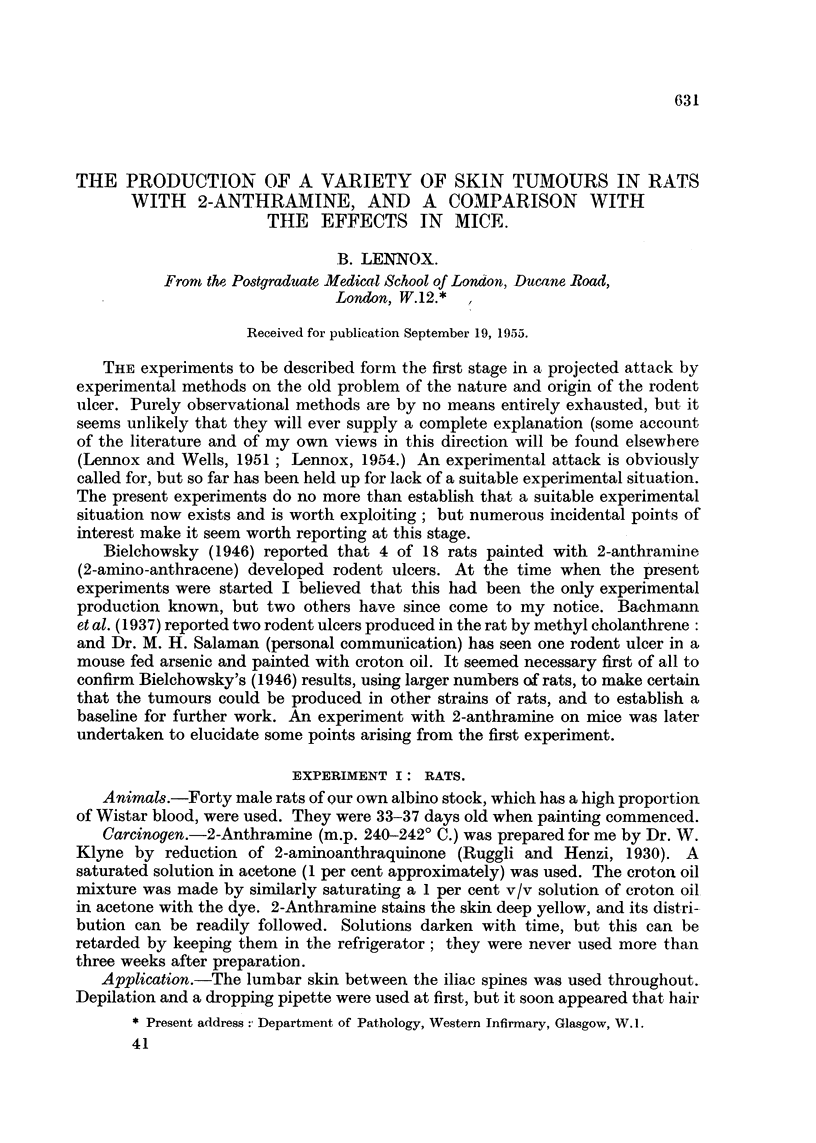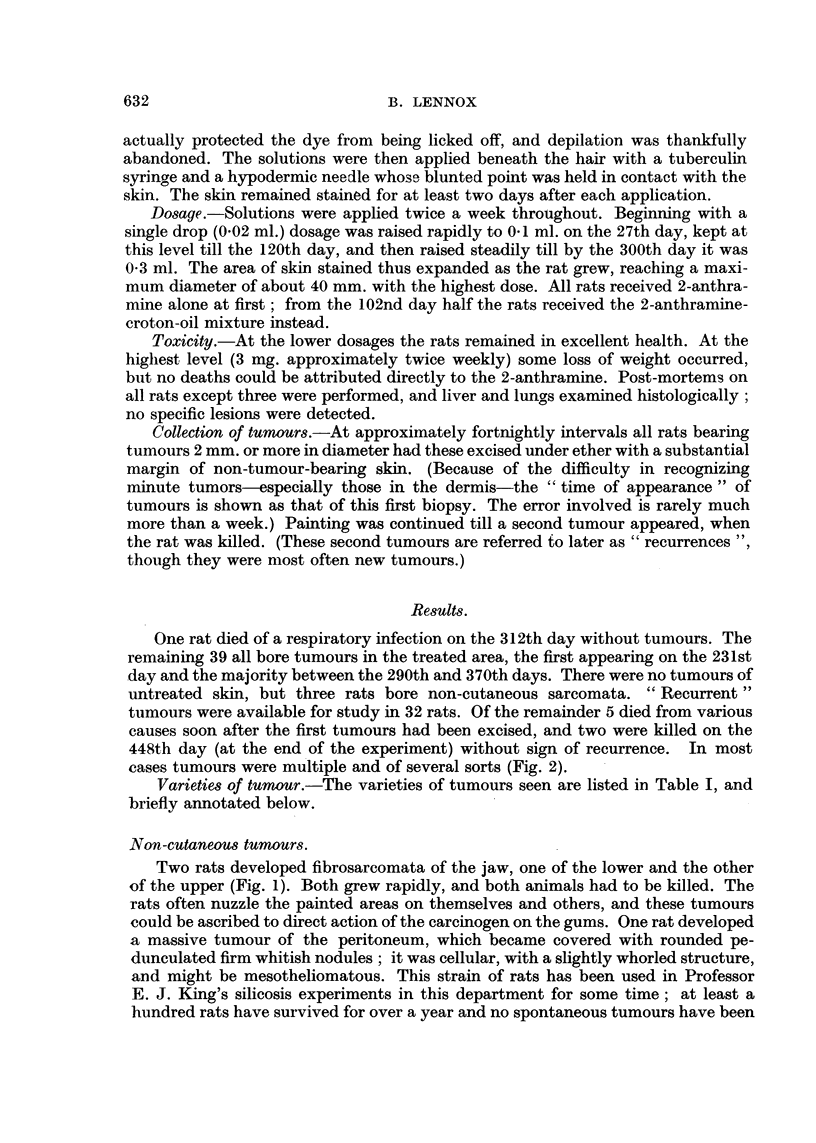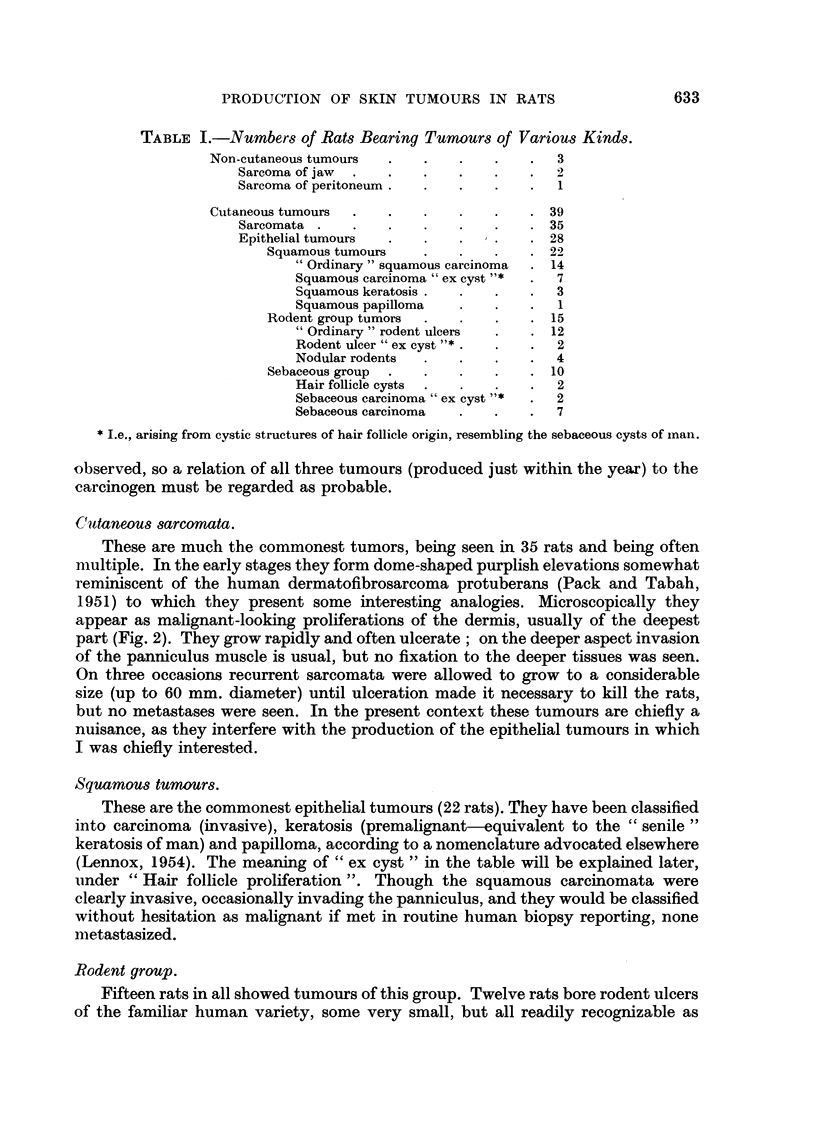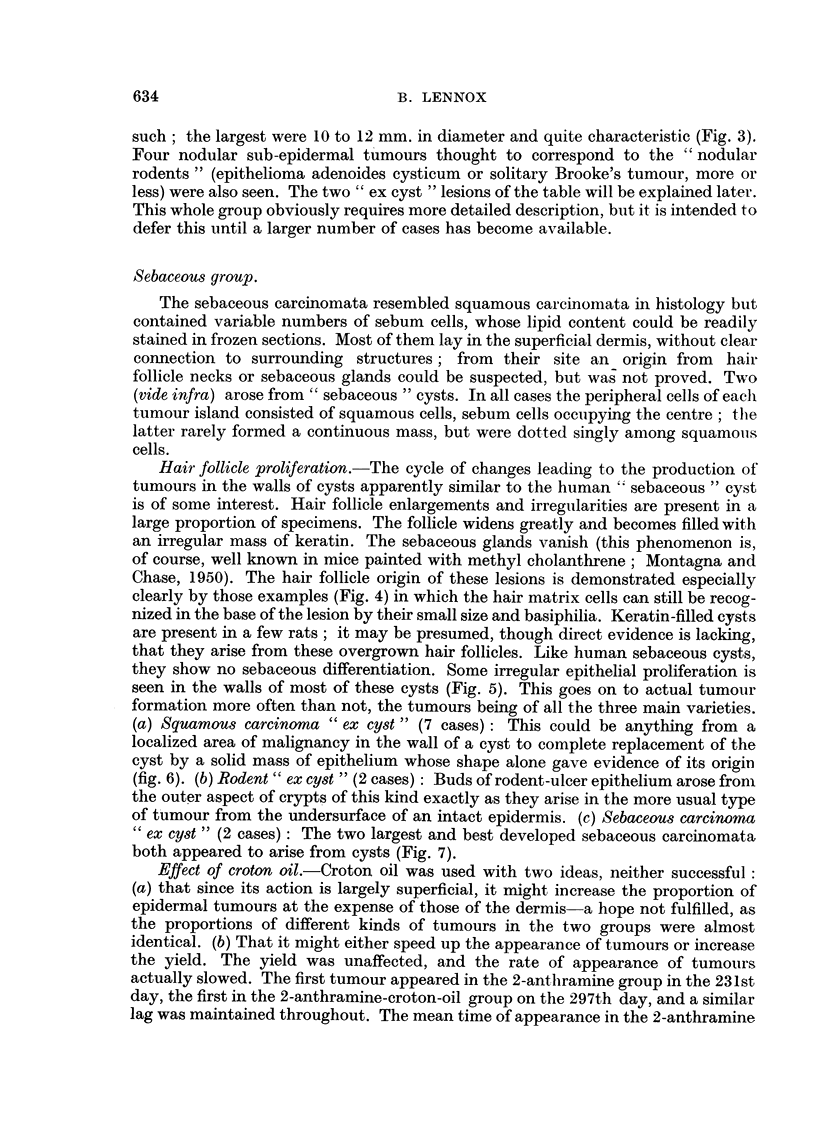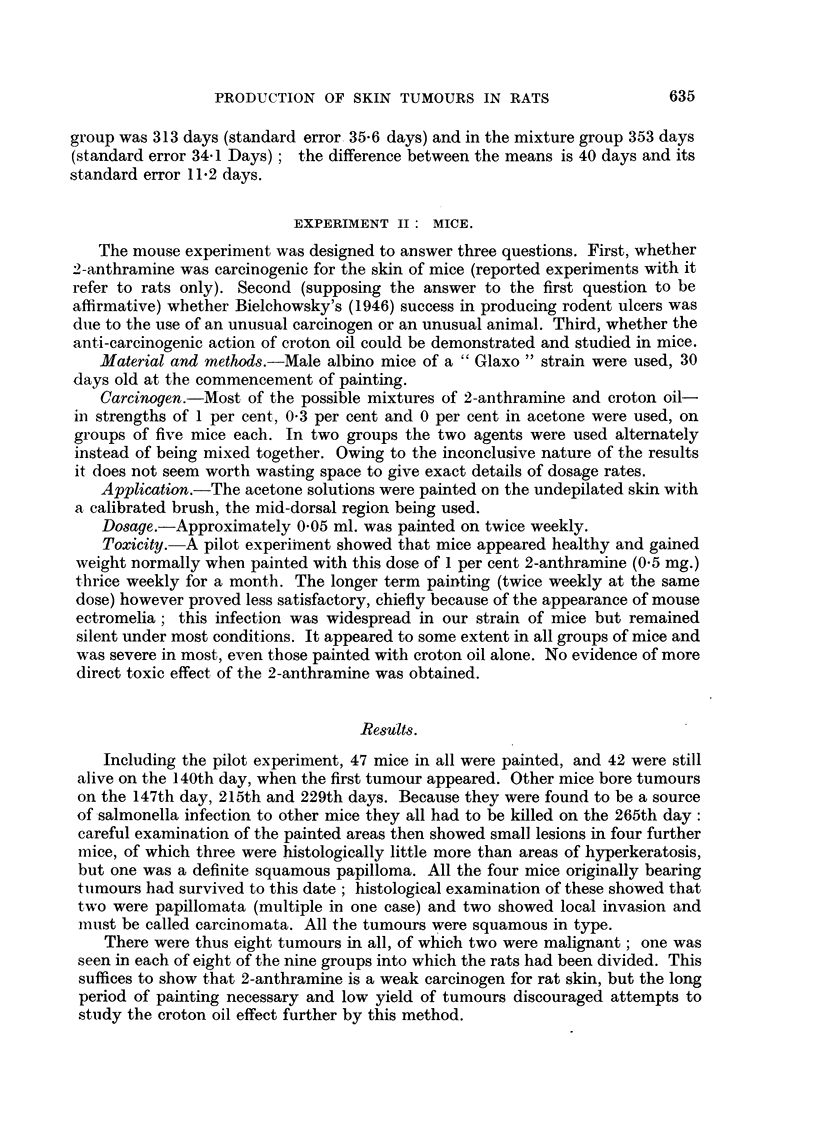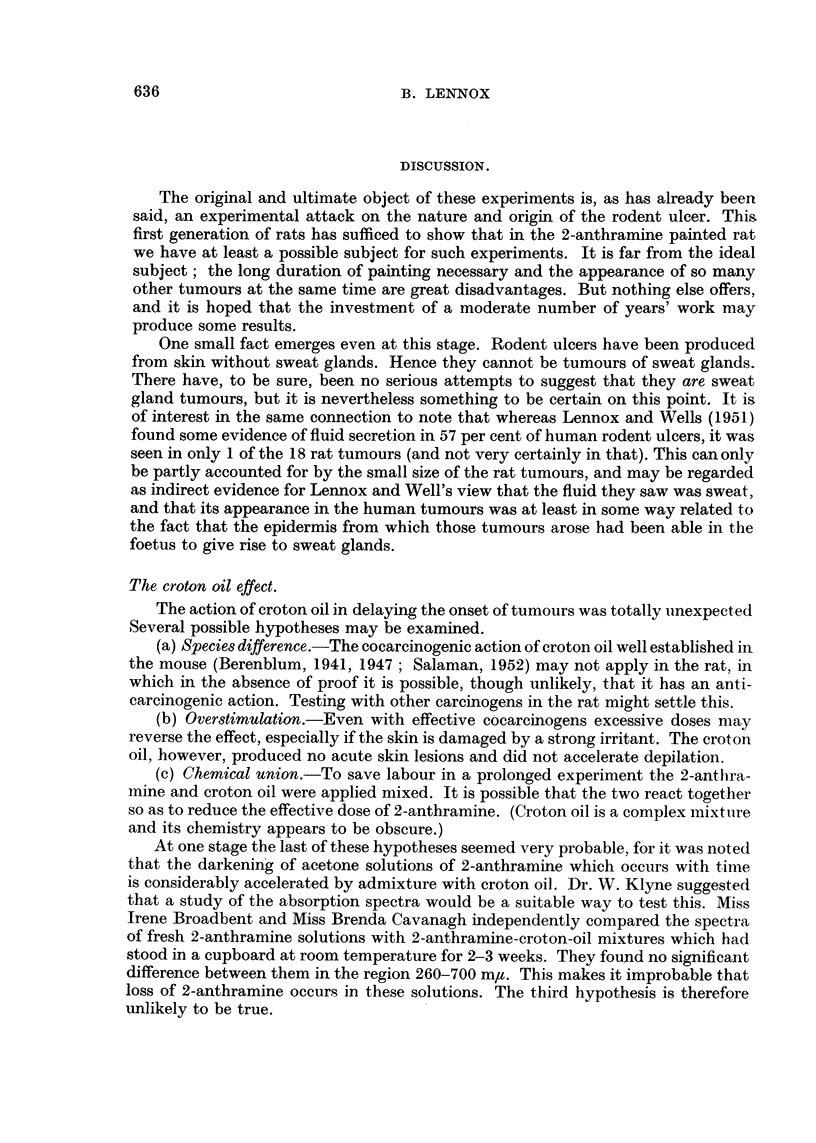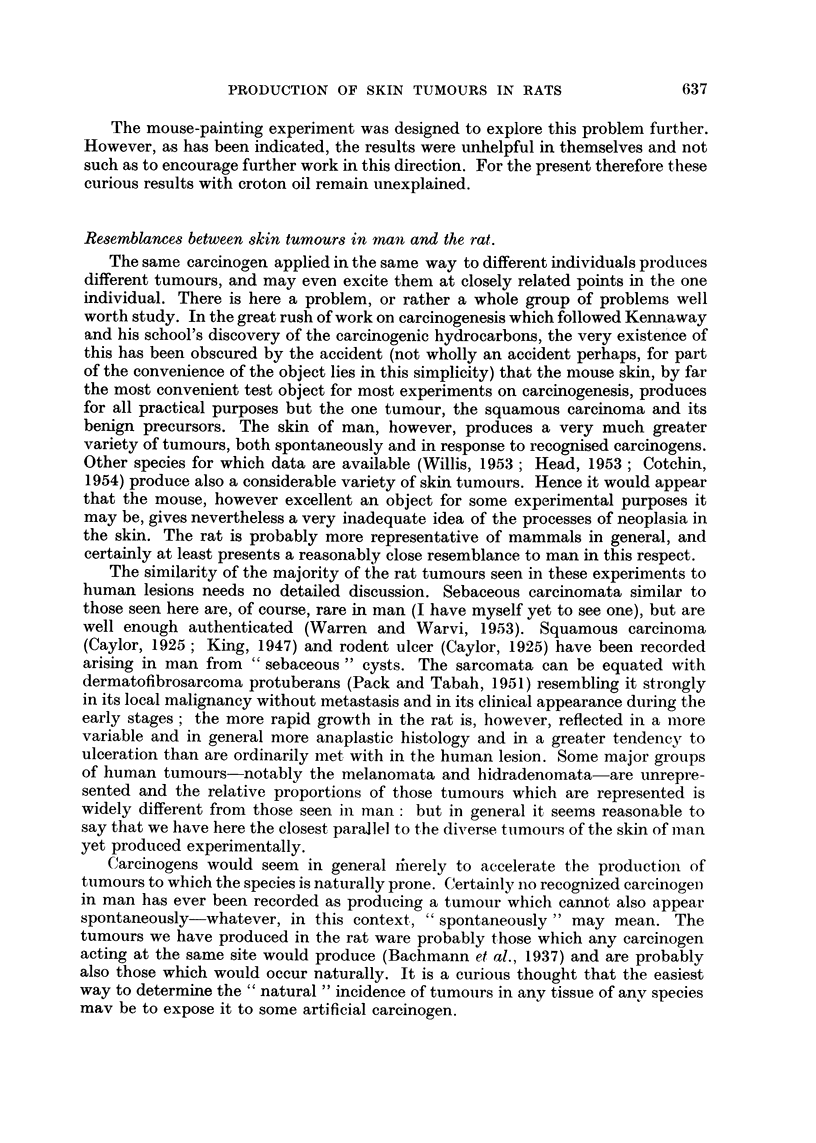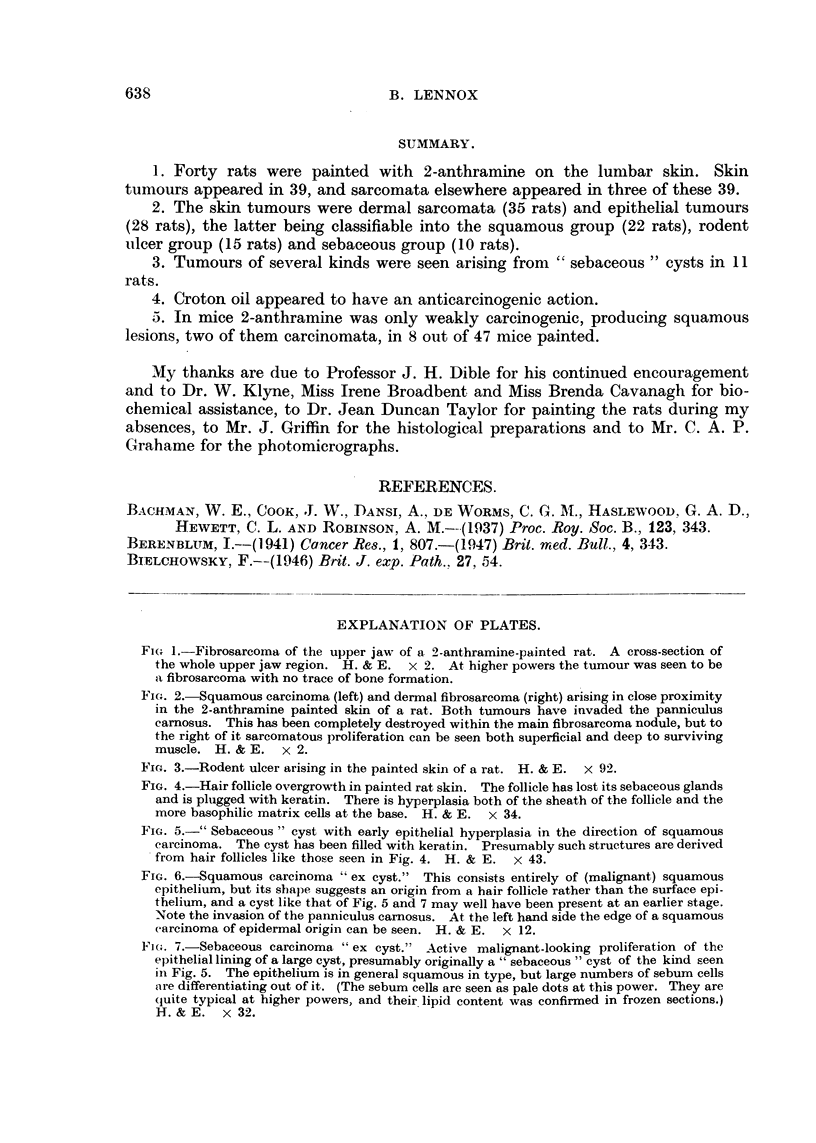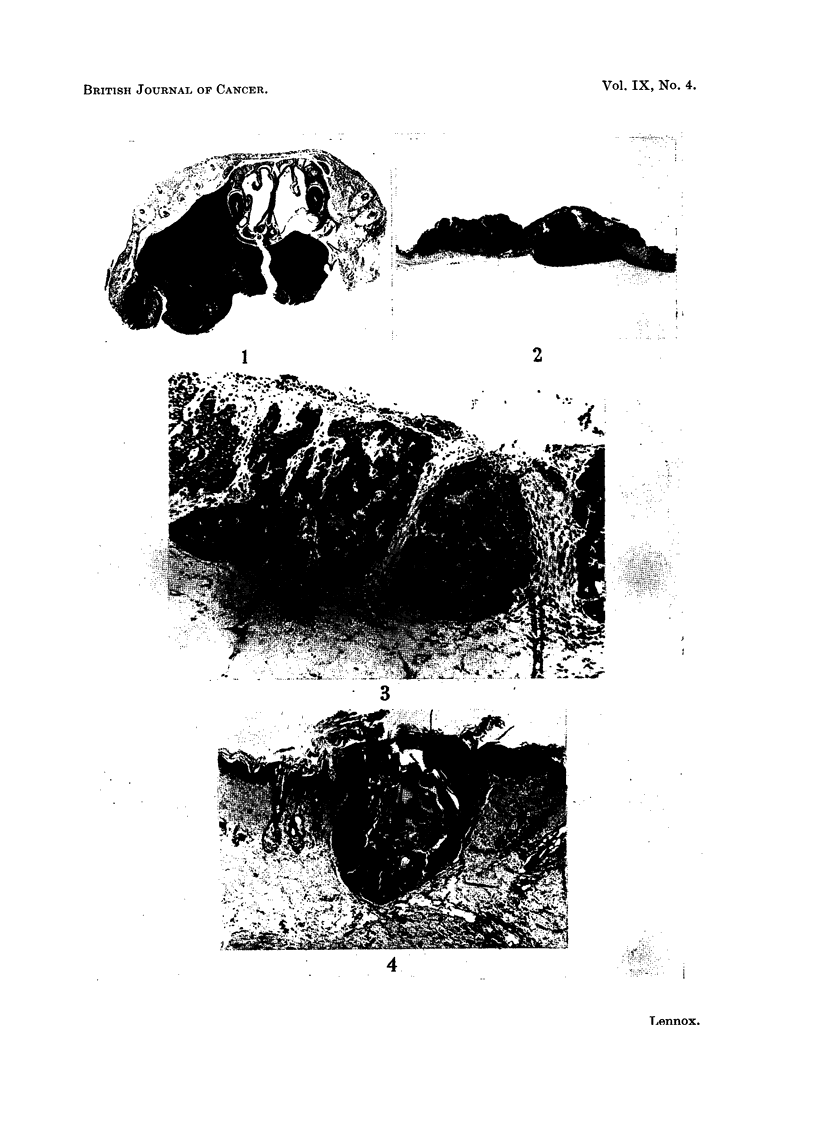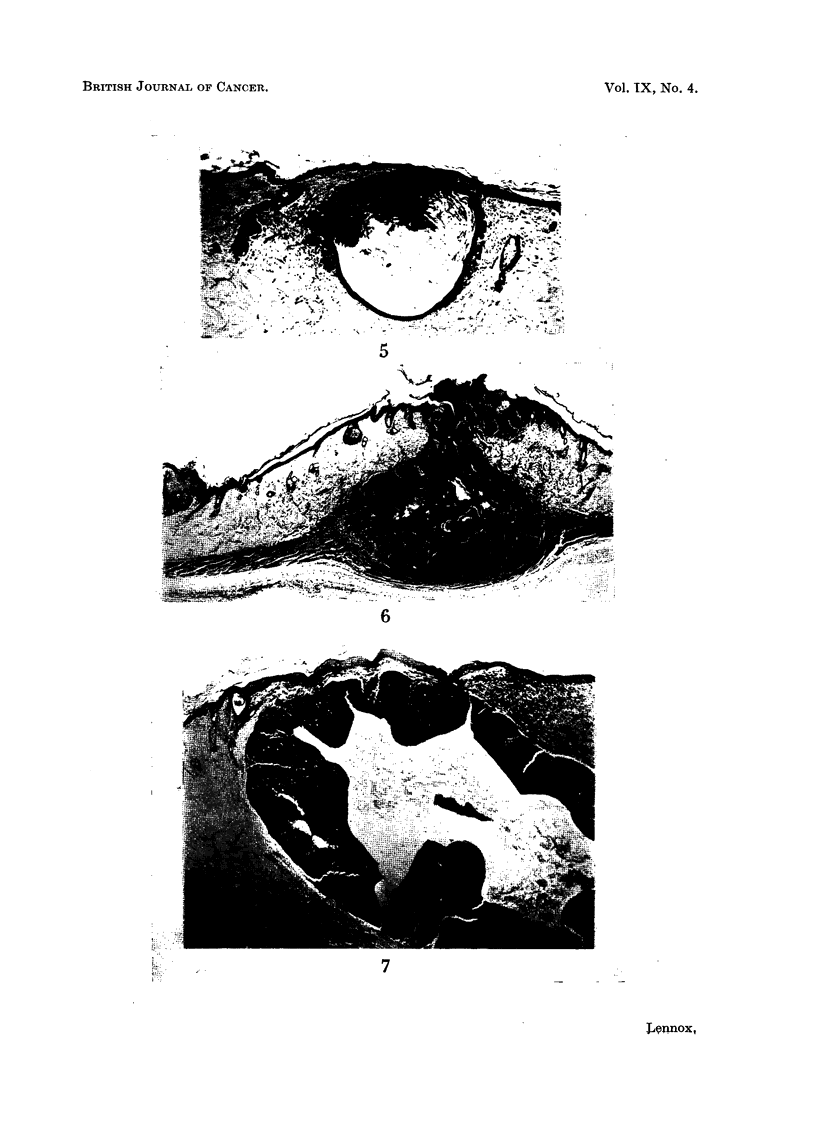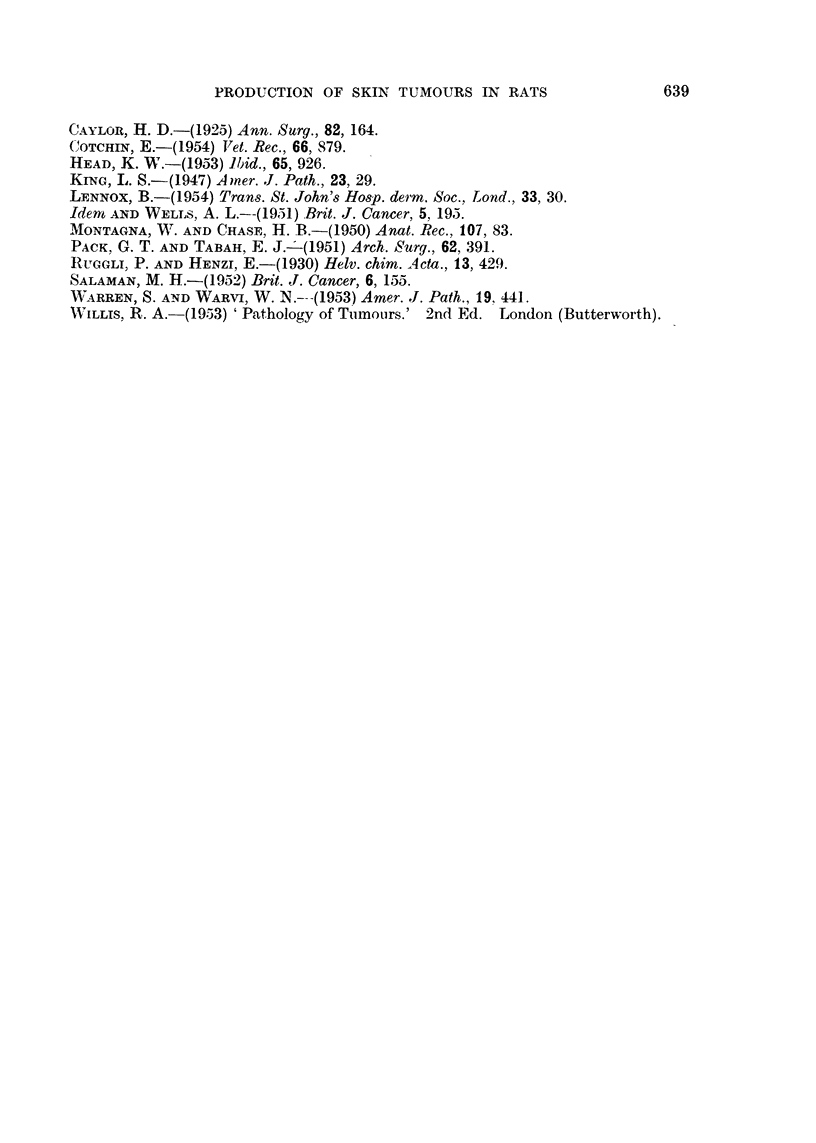# The Production of a Variety of Skin Tumours in Rats with 2-Anthramine, and a Comparison with the Effects in Mice

**DOI:** 10.1038/bjc.1955.67

**Published:** 1955-12

**Authors:** B. Lennox

## Abstract

**Images:**


### THE PRODUCTION OF A VARIETY OF SKIN TUMOURS IN RATS WITH 2-ANTHRAMINE, AND A COMPARISON WITH THE EFFECTS IN MICE.

B. LENNOX.

*From the Postgraduate Medical School of London, Ducane Road, London, W.12.**

Received for publication September 19, 1955.

THE experiments to be described form the first stage in a projected attack by experimental methods on the old problem of the nature and origin of the rodent ulcer. Purely observational methods are by no means entirely exhausted, but it seems unlikely that they will ever supply a complete explanation (some account of the literature and of my own views in this direction will be found elsewhere (Lennox and Wells, 1951 ; Lennox, 1954.) An experimental attack is obviously called for, but so far has been held up for lack of a suitable experimental situation. The present experiments do no more than establish that a suitable experimental situation now exists and is worth exploiting ; but numerous incidental points of interest make it seem worth reporting at this stage.

Bielchowsky (1946) reported that 4 of 18 rats painted with 2-anthramine (2-amino-anthracene) developed rodent ulcers. At the time when the present experiments were started I believed that this had been the only experimental production known, but two others have since come to my notice. Bachmann *et al.* (1937) reported two rodent ulcers produced in the rat by methyl cholanthrene : and Dr. M. H. Salaman (personal communication) has seen one rodent ulcer in a mouse fed arsenic and painted with croton oil. It seemed necessary first of all to confirm Bielchowsky's (1946) results, using larger numbers of rats, to make certain that the tumours could be produced in other strains of rats, and to establish a baseline for further work. An experiment with 2-anthramine on mice was later undertaken to elucidate some points arising from the first experiment.

### EXPERIMENT I: RATS.

*Animals.*—Forty male rats of our own albino stock, which has a high proportion of Wistar blood, were used. They were 33–37 days old when painting commenced.

*Carcinogen.*—2-Anthramine (m.p. 240–242° C.) was prepared for me by Dr. W. Klyne by reduction of 2-aminoanthraquinone (Ruggli and Henzi, 1930). A saturated solution in acetone (1 per cent approximately) was used. The croton oil mixture was made by similarly saturating a 1 per cent v/v solution of croton oil in acetone with the dye. 2-Anthramine stains the skin deep yellow, and its distribution can be readily followed. Solutions darken with time, but this can be retarded by keeping them in the refrigerator ; they were never used more than three weeks after preparation.

*Application.*—The lumbar skin between the iliac spines was used throughout. Depilation and a dropping pipette were used at first, but it soon appeared that hair

* Present address : Department of Pathology, Western Infirmary, Glasgow, W.1.

actually protected the dye from being licked off, and depilation was thankfully abandoned. The solutions were then applied beneath the hair with a tuberculin syringe and a hypodermic needle whose blunted point was held in contact with the skin. The skin remained stained for at least two days after each application.

*Dosage*.—Solutions were applied twice a week throughout. Beginning with a single drop (0·02 ml.) dosage was raised rapidly to 0·1 ml. on the 27th day, kept at this level till the 120th day, and then raised steadily till by the 300th day it was 0·3 ml. The area of skin stained thus expanded as the rat grew, reaching a maximum diameter of about 40 mm. with the highest dose. All rats received 2-anthramine alone at first ; from the 102nd day half the rats received the 2-anthramine-croton-oil mixture instead.

*Toxicity*.—At the lower dosages the rats remained in excellent health. At the highest level (3 mg. approximately twice weekly) some loss of weight occurred, but no deaths could be attributed directly to the 2-anthramine. Post-mortems on all rats except three were performed, and liver and lungs examined histologically ; no specific lesions were detected.

*Collection of tumours*.—At approximately fortnightly intervals all rats bearing tumours 2 mm. or more in diameter had these excised under ether with a substantial margin of non-tumour-bearing skin. (Because of the difficulty in recognizing minute tumors—especially those in the dermis—the "time of appearance" of tumours is shown as that of this first biopsy. The error involved is rarely much more than a week.) Painting was continued till a second tumour appeared, when the rat was killed. (These second tumours are referred to later as "recurrences", though they were most often new tumours.)

### *Results.*

One rat died of a respiratory infection on the 312th day without tumours. The remaining 39 all bore tumours in the treated area, the first appearing on the 231st day and the majority between the 290th and 370th days. There were no tumours of untreated skin, but three rats bore non-cutaneous sarcomata. "Recurrent" tumours were available for study in 32 rats. Of the remainder 5 died from various causes soon after the first tumours had been excised, and two were killed on the 448th day (at the end of the experiment) without sign of recurrence. In most cases tumours were multiple and of several sorts (Fig. 2).

*Varieties of tumour*.—The varieties of tumours seen are listed in Table I, and briefly annotated below.

### *Non-cutaneous tumours.*

Two rats developed fibrosarcomata of the jaw, one of the lower and the other of the upper (Fig. 1). Both grew rapidly, and both animals had to be killed. The rats often nuzzle the painted areas on themselves and others, and these tumours could be ascribed to direct action of the carcinogen on the gums. One rat developed a massive tumour of the peritoneum, which became covered with rounded pedunculated firm whitish nodules ; it was cellular, with a slightly whorled structure, and might be mesotheliomatous. This strain of rats has been used in Professor E. J. King's silicosis experiments in this department for some time ; at least a hundred rats have survived for over a year and no spontaneous tumours have been

TABLE I.—*Numbers of Rats Bearing Tumours of Various Kinds.*

| | |
|---|---|
| Non-cutaneous tumours | 3 |
| Sarcoma of jaw | 2 |
| Sarcoma of peritoneum | 1 |
| | |
| Cutaneous tumours | 39 |
| Sarcomata | 35 |
| Epithelial tumours | 28 |
| Squamous tumours | 22 |
| " Ordinary " squamous carcinoma | 14 |
| Squamous carcinoma " ex cyst "* | 7 |
| Squamous keratosis | 3 |
| Squamous papilloma | 1 |
| Rodent group tumors | 15 |
| " Ordinary " rodent ulcers | 12 |
| Rodent ulcer " ex cyst "* | 2 |
| Nodular rodents | 4 |
| Sebaceous group | 10 |
| Hair follicle cysts | 2 |
| Sebaceous carcinoma " ex cyst "* | 2 |
| Sebaceous carcinoma | 7 |

* I.e., arising from cystic structures of hair follicle origin, resembling the sebaceous cysts of man.

observed, so a relation of all three tumours (produced just within the year) to the carcinogen must be regarded as probable.

*Cutaneous sarcomata.*

These are much the commonest tumors, being seen in 35 rats and being often multiple. In the early stages they form dome-shaped purplish elevations somewhat reminiscent of the human dermatofibrosarcoma protuberans (Pack and Tabah, 1951) to which they present some interesting analogies. Microscopically they appear as malignant-looking proliferations of the dermis, usually of the deepest part (Fig. 2). They grow rapidly and often ulcerate ; on the deeper aspect invasion of the panniculus muscle is usual, but no fixation to the deeper tissues was seen. On three occasions recurrent sarcomata were allowed to grow to a considerable size (up to 60 mm. diameter) until ulceration made it necessary to kill the rats, but no metastases were seen. In the present context these tumours are chiefly a nuisance, as they interfere with the production of the epithelial tumours in which I was chiefly interested.

*Squamous tumours.*

These are the commonest epithelial tumours (22 rats). They have been classified into carcinoma (invasive), keratosis (premalignant—equivalent to the " senile " keratosis of man) and papilloma, according to a nomenclature advocated elsewhere (Lennox, 1954). The meaning of " ex cyst " in the table will be explained later, under " Hair follicle proliferation ". Though the squamous carcinomata were clearly invasive, occasionally invading the panniculus, and they would be classified without hesitation as malignant if met in routine human biopsy reporting, none metastasized.

*Rodent group.*

Fifteen rats in all showed tumours of this group. Twelve rats bore rodent ulcers of the familiar human variety, some very small, but all readily recognizable as such ; the largest were 10 to 12 mm. in diameter and quite characteristic (Fig. 3). Four nodular sub-epidermal tumours thought to correspond to the " nodular rodents " (epithelioma adenoides cysticum or solitary Brooke's tumour, more or less) were also seen. The two " ex cyst " lesions of the table will be explained later. This whole group obviously requires more detailed description, but it is intended to defer this until a larger number of cases has become available.

*Sebaceous group.*

The sebaceous carcinomata resembled squamous carcinomata in histology but contained variable numbers of sebum cells, whose lipid content could be readily stained in frozen sections. Most of them lay in the superficial dermis, without clear connection to surrounding structures ; from their site an origin from hair follicle necks or sebaceous glands could be suspected, but was not proved. Two (*vide infra*) arose from " sebaceous " cysts. In all cases the peripheral cells of each tumour island consisted of squamous cells, sebum cells occupying the centre ; the latter rarely formed a continuous mass, but were dotted singly among squamous cells.

*Hair follicle proliferation.*—The cycle of changes leading to the production of tumours in the walls of cysts apparently similar to the human " sebaceous " cyst is of some interest. Hair follicle enlargements and irregularities are present in a large proportion of specimens. The follicle widens greatly and becomes filled with an irregular mass of keratin. The sebaceous glands vanish (this phenomenon is, of course, well known in mice painted with methyl cholanthrene ; Montagna and Chase, 1950). The hair follicle origin of these lesions is demonstrated especially clearly by those examples (Fig. 4) in which the hair matrix cells can still be recognized in the base of the lesion by their small size and basiphilia. Keratin-filled cysts are present in a few rats ; it may be presumed, though direct evidence is lacking, that they arise from these overgrown hair follicles. Like human sebaceous cysts, they show no sebaceous differentiation. Some irregular epithelial proliferation is seen in the walls of most of these cysts (Fig. 5). This goes on to actual tumour formation more often than not, the tumours being of all the three main varieties. (*a*) *Squamous carcinoma " ex cyst "* (7 cases) : This could be anything from a localized area of malignancy in the wall of a cyst to complete replacement of the cyst by a solid mass of epithelium whose shape alone gave evidence of its origin (fig. 6). (*b*) *Rodent " ex cyst "* (2 cases) : Buds of rodent-ulcer epithelium arose from the outer aspect of crypts of this kind exactly as they arise in the more usual type of tumour from the undersurface of an intact epidermis. (*c*) *Sebaceous carcinoma " ex cyst "* (2 cases) : The two largest and best developed sebaceous carcinomata both appeared to arise from cysts (Fig. 7).

*Effect of croton oil.*—Croton oil was used with two ideas, neither successful : (*a*) that since its action is largely superficial, it might increase the proportion of epidermal tumours at the expense of those of the dermis—a hope not fulfilled, as the proportions of different kinds of tumours in the two groups were almost identical. (*b*) That it might either speed up the appearance of tumours or increase the yield. The yield was unaffected, and the rate of appearance of tumours actually slowed. The first tumour appeared in the 2-anthramine group in the 231st day, the first in the 2-anthramine-croton-oil group on the 297th day, and a similar lag was maintained throughout. The mean time of appearance in the 2-anthramine group was 313 days (standard error 35·6 days) and in the mixture group 353 days (standard error 34·1 Days); the difference between the means is 40 days and its standard error 11·2 days.

EXPERIMENT II: MICE.

The mouse experiment was designed to answer three questions. First, whether 2-anthramine was carcinogenic for the skin of mice (reported experiments with it refer to rats only). Second (supposing the answer to the first question to be affirmative) whether Bielchowsky's (1946) success in producing rodent ulcers was due to the use of an unusual carcinogen or an unusual animal. Third, whether the anti-carcinogenic action of croton oil could be demonstrated and studied in mice.

*Material and methods.*—Male albino mice of a "Glaxo" strain were used, 30 days old at the commencement of painting.

*Carcinogen.*—Most of the possible mixtures of 2-anthramine and croton oil—in strengths of 1 per cent, 0·3 per cent and 0 per cent in acetone were used, on groups of five mice each. In two groups the two agents were used alternately instead of being mixed together. Owing to the inconclusive nature of the results it does not seem worth wasting space to give exact details of dosage rates.

*Application.*—The acetone solutions were painted on the undepilated skin with a calibrated brush, the mid-dorsal region being used.

*Dosage.*—Approximately 0·05 ml. was painted on twice weekly.

*Toxicity.*—A pilot experiment showed that mice appeared healthy and gained weight normally when painted with this dose of 1 per cent 2-anthramine (0·5 mg.) thrice weekly for a month. The longer term painting (twice weekly at the same dose) however proved less satisfactory, chiefly because of the appearance of mouse ectromelia; this infection was widespread in our strain of mice but remained silent under most conditions. It appeared to some extent in all groups of mice and was severe in most, even those painted with croton oil alone. No evidence of more direct toxic effect of the 2-anthramine was obtained.

*Results.*

Including the pilot experiment, 47 mice in all were painted, and 42 were still alive on the 140th day, when the first tumour appeared. Other mice bore tumours on the 147th day, 215th and 229th days. Because they were found to be a source of salmonella infection to other mice they all had to be killed on the 265th day: careful examination of the painted areas then showed small lesions in four further mice, of which three were histologically little more than areas of hyperkeratosis, but one was a definite squamous papilloma. All the four mice originally bearing tumours had survived to this date; histological examination of these showed that two were papillomata (multiple in one case) and two showed local invasion and must be called carcinomata. All the tumours were squamous in type.

There were thus eight tumours in all, of which two were malignant; one was seen in each of eight of the nine groups into which the rats had been divided. This suffices to show that 2-anthramine is a weak carcinogen for rat skin, but the long period of painting necessary and low yield of tumours discouraged attempts to study the croton oil effect further by this method.

### DISCUSSION.

The original and ultimate object of these experiments is, as has already been said, an experimental attack on the nature and origin of the rodent ulcer. This first generation of rats has sufficed to show that in the 2-anthramine painted rat we have at least a possible subject for such experiments. It is far from the ideal subject ; the long duration of painting necessary and the appearance of so many other tumours at the same time are great disadvantages. But nothing else offers, and it is hoped that the investment of a moderate number of years' work may produce some results.

One small fact emerges even at this stage. Rodent ulcers have been produced from skin without sweat glands. Hence they cannot be tumours of sweat glands. There have, to be sure, been no serious attempts to suggest that they *are* sweat gland tumours, but it is nevertheless something to be certain on this point. It is of interest in the same connection to note that whereas Lennox and Wells (1951) found some evidence of fluid secretion in 57 per cent of human rodent ulcers, it was seen in only 1 of the 18 rat tumours (and not very certainly in that). This can only be partly accounted for by the small size of the rat tumours, and may be regarded as indirect evidence for Lennox and Well's view that the fluid they saw was sweat, and that its appearance in the human tumours was at least in some way related to the fact that the epidermis from which those tumours arose had been able in the foetus to give rise to sweat glands.

*The croton oil effect.*

The action of croton oil in delaying the onset of tumours was totally unexpected Several possible hypotheses may be examined.

(a) *Species difference.*—The cocarcinogenic action of croton oil well established in the mouse (Berenblum, 1941, 1947 ; Salaman, 1952) may not apply in the rat, in which in the absence of proof it is possible, though unlikely, that it has an anti-carcinogenic action. Testing with other carcinogens in the rat might settle this.

(b) *Overstimulation.*—Even with effective cocarcinogens excessive doses may reverse the effect, especially if the skin is damaged by a strong irritant. The croton oil, however, produced no acute skin lesions and did not accelerate depilation.

(c) *Chemical union.*—To save labour in a prolonged experiment the 2-anthramine and croton oil were applied mixed. It is possible that the two react together so as to reduce the effective dose of 2-anthramine. (Croton oil is a complex mixture and its chemistry appears to be obscure.)

At one stage the last of these hypotheses seemed very probable, for it was noted that the darkening of acetone solutions of 2-anthramine which occurs with time is considerably accelerated by admixture with croton oil. Dr. W. Klyne suggested that a study of the absorption spectra would be a suitable way to test this. Miss Irene Broadbent and Miss Brenda Cavanagh independently compared the spectra of fresh 2-anthramine solutions with 2-anthramine-croton-oil mixtures which had stood in a cupboard at room temperature for 2–3 weeks. They found no significant difference between them in the region 260–700 m$\mu$. This makes it improbable that loss of 2-anthramine occurs in these solutions. The third hypothesis is therefore unlikely to be true.

The mouse-painting experiment was designed to explore this problem further. However, as has been indicated, the results were unhelpful in themselves and not such as to encourage further work in this direction. For the present therefore these curious results with croton oil remain unexplained.

*Resemblances between skin tumours in man and the rat.*

The same carcinogen applied in the same way to different individuals produces different tumours, and may even excite them at closely related points in the one individual. There is here a problem, or rather a whole group of problems well worth study. In the great rush of work on carcinogenesis which followed Kennaway and his school's discovery of the carcinogenic hydrocarbons, the very existence of this has been obscured by the accident (not wholly an accident perhaps, for part of the convenience of the object lies in this simplicity) that the mouse skin, by far the most convenient test object for most experiments on carcinogenesis, produces for all practical purposes but the one tumour, the squamous carcinoma and its benign precursors. The skin of man, however, produces a very much greater variety of tumours, both spontaneously and in response to recognised carcinogens. Other species for which data are available (Willis, 1953 ; Head, 1953 ; Cotchin, 1954) produce also a considerable variety of skin tumours. Hence it would appear that the mouse, however excellent an object for some experimental purposes it may be, gives nevertheless a very inadequate idea of the processes of neoplasia in the skin. The rat is probably more representative of mammals in general, and certainly at least presents a reasonably close resemblance to man in this respect.

The similarity of the majority of the rat tumours seen in these experiments to human lesions needs no detailed discussion. Sebaceous carcinomata similar to those seen here are, of course, rare in man (I have myself yet to see one), but are well enough authenticated (Warren and Warvi, 1953). Squamous carcinoma (Caylor, 1925 ; King, 1947) and rodent ulcer (Caylor, 1925) have been recorded arising in man from " sebaceous " cysts. The sarcomata can be equated with dermatofibrosarcoma protuberans (Pack and Tabah, 1951) resembling it strongly in its local malignancy without metastasis and in its clinical appearance during the early stages ; the more rapid growth in the rat is, however, reflected in a more variable and in general more anaplastic histology and in a greater tendency to ulceration than are ordinarily met with in the human lesion. Some major groups of human tumours—notably the melanomata and hidradenomata—are unrepresented and the relative proportions of those tumours which are represented is widely different from those seen in man : but in general it seems reasonable to say that we have here the closest parallel to the diverse tumours of the skin of man yet produced experimentally.

Carcinogens would seem in general merely to accelerate the production of tumours to which the species is naturally prone. Certainly no recognized carcinogen in man has ever been recorded as producing a tumour which cannot also appear spontaneously—whatever, in this context, " spontaneously " may mean. The tumours we have produced in the rat ware probably those which any carcinogen acting at the same site would produce (Bachmann *et al.*, 1937) and are probably also those which would occur naturally. It is a curious thought that the easiest way to determine the " natural " incidence of tumours in any tissue of any species mav be to expose it to some artificial carcinogen.

SUMMARY.

1. Forty rats were painted with 2-anthramine on the lumbar skin. Skin tumours appeared in 39, and sarcomata elsewhere appeared in three of these 39.

2. The skin tumours were dermal sarcomata (35 rats) and epithelial tumours (28 rats), the latter being classifiable into the squamous group (22 rats), rodent ulcer group (15 rats) and sebaceous group (10 rats).

3. Tumours of several kinds were seen arising from " sebaceous " cysts in 11 rats.

4. Croton oil appeared to have an anticarcinogenic action.

5. In mice 2-anthramine was only weakly carcinogenic, producing squamous lesions, two of them carcinomata, in 8 out of 47 mice painted.

My thanks are due to Professor J. H. Dible for his continued encouragement and to Dr. W. Klyne, Miss Irene Broadbent and Miss Brenda Cavanagh for biochemical assistance, to Dr. Jean Duncan Taylor for painting the rats during my absences, to Mr. J. Griffin for the histological preparations and to Mr. C. A. P. Grahame for the photomicrographs.

### REFERENCES.

Bachman, W. E., Cook, J. W., Dansi, A., de Worms, C. G. M., Haslewood, G. A. D., Hewett, C. L. and Robinson, A. M.—(1937) *Proc. Roy. Soc.* B., **123**, 343.

Berenblum, I.—(1941) *Cancer Res.*, **1**, 807.—(1947) *Brit. med. Bull.*, **4**, 343.

Bielchowsky, F.—(1946) *Brit. J. exp. Path.*, **27**, 54.

### EXPLANATION OF PLATES.

Fig 1.—Fibrosarcoma of the upper jaw of a 2-anthramine-painted rat. A cross-section of the whole upper jaw region. H. & E. × 2. At higher powers the tumour was seen to be a fibrosarcoma with no trace of bone formation.

Fig. 2.—Squamous carcinoma (left) and dermal fibrosarcoma (right) arising in close proximity in the 2-anthramine painted skin of a rat. Both tumours have invaded the panniculus carnosus. This has been completely destroyed within the main fibrosarcoma nodule, but to the right of it sarcomatous proliferation can be seen both superficial and deep to surviving muscle. H. & E. × 2.

Fig. 3.—Rodent ulcer arising in the painted skin of a rat. H. & E. × 92.

Fig. 4.—Hair follicle overgrowth in painted rat skin. The follicle has lost its sebaceous glands and is plugged with keratin. There is hyperplasia both of the sheath of the follicle and the more basophilic matrix cells at the base. H. & E. × 34.

Fig. 5.—" Sebaceous " cyst with early epithelial hyperplasia in the direction of squamous carcinoma. The cyst has been filled with keratin. Presumably such structures are derived from hair follicles like those seen in Fig. 4. H. & E. × 43.

Fig. 6.—Squamous carcinoma " ex cyst." This consists entirely of (malignant) squamous epithelium, but its shape suggests an origin from a hair follicle rather than the surface epithelium, and a cyst like that of Fig. 5 and 7 may well have been present at an earlier stage. Note the invasion of the panniculus carnosus. At the left hand side the edge of a squamous carcinoma of epidermal origin can be seen. H. & E. × 12.

Fig. 7.—Sebaceous carcinoma " ex cyst." Active malignant-looking proliferation of the epithelial lining of a large cyst, presumably originally a " sebaceous " cyst of the kind seen in Fig. 5. The epithelium is in general squamous in type, but large numbers of sebum cells are differentiating out of it. (The sebum cells are seen as pale dots at this power. They are quite typical at higher powers, and their lipid content was confirmed in frozen sections.) H. & E. × 32.

1

2

3

4

5

6

7

CAYLOR, H. D.—(1925) *Ann. Surg.*, **82**, 164.
COTCHIN, E.—(1954) *Vet. Rec.*, **66**, 879.
HEAD, K. W.—(1953) *Ibid.*, **65**, 926.
KING, L. S.—(1947) *Amer. J. Path.*, **23**, 29.
LENNOX, B.—(1954) *Trans. St. John's Hosp. derm. Soc., Lond.*, **33**, 30.
*Idem* AND WELLS, A. L.—(1951) *Brit. J. Cancer*, **5**, 195.
MONTAGNA, W. AND CHASE, H. B.—(1950) *Anat. Rec.*, **107**, 83.
PACK, G. T. AND TABAH, E. J.—(1951) *Arch. Surg.*, **62**, 391.
RUGGLI, P. AND HENZI, E.—(1930) *Helv. chim. Acta.*, **13**, 429.
SALAMAN, M. H.—(1952) *Brit. J. Cancer*, **6**, 155.
WARREN, S. AND WARVI, W. N.—(1953) *Amer. J. Path.*, **19**, 441.
WILLIS, R. A.—(1953) 'Pathology of Tumours.' 2nd Ed. London (Butterworth).